# Precision synbiotics increase gut microbiome diversity and improve gastrointestinal symptoms in a pilot open-label study for autism spectrum disorder

**DOI:** 10.1128/msystems.00503-24

**Published:** 2024-04-25

**Authors:** Joann Phan, Diana C. Calvo, Divya Nair, Suneer Jain, Thibaut Montagne, Summer Dietsche, Kelsey Blanchard, Shirin Treadwell, James Adams, Rosa Krajmalnik-Brown

**Affiliations:** 1 Sun Genomics, Inc., San Diego, California, USA; 2 Department of Civil Engineering, Construction Management, and Environmental Engineering, Northern Arizona University, Flagstaff, Arizona, USA; 3 Biodesign Center for Health Through Microbiomes, Arizona State University, Tempe, Arizona, USA; Argonne National Laboratory, Lemont, Illinois, USA

**Keywords:** ASD, synbiotics, probiotics, prebiotics, gut microbiome, open-label, precision, supplements, metagenomics

## Abstract

The efficacy of prebiotics and probiotics (synbiotics when combined) to improve symptoms associated with autism spectrum disorder (ASD) has shown considerable inter-study variation, likely due to the complex, heterogeneous nature of the disorder and its associated behavioral, developmental, and gastrointestinal symptoms. Here, we present a precision synbiotic supplementation study in 296 children and adults diagnosed with ASD versus 123 age-matched neurotypical controls. One hundred seventy ASD participants completed the study. Baseline and post-synbiotic assessment of ASD and gastrointestinal (GI) symptoms and deep metagenomic sequencing were performed. Within the ASD cohort, there were significant differences in microbes between subpopulations based on the social responsiveness scale (SRS2) survey (*Prevotella* spp.*, Bacteroides, Fusicatenibacter*, and others) and gluten and dairy-free diets (*Bifidobacterium* spp., *Lactococcus, Streptococcus* spp., and others). At the baseline, the ASD cohort maintained a lower taxonomic alpha diversity and significant differences in taxonomic composition, metabolic pathways, and gene families, with a greater proportion of potential pathogens, including *Shigella, Klebsiella,* and *Clostridium,* and lower proportions of beneficial microbes, including *Faecalibacterium* compared to controls. Following the 3-month synbiotic supplementation, the ASD cohort showed increased taxonomic alpha diversity, shifts in taxonomy and metabolic pathway potential, and improvements in some ASD-related symptoms, including a significant reduction in GI discomfort and overall improved language, comprehension, cognition, thinking, and speech. However, the open-label study design may include some placebo effects. In summary, we found that precision synbiotics modulated the gut microbiome and could be used as supplementation to improve gastrointestinal and ASD-related symptoms.

**IMPORTANCE:**

Autism spectrum disorder (ASD) is prevalent in 1 out of 36 children in the United States and contributes to health, financial, and psychological burdens. Attempts to identify a gut microbiome signature of ASD have produced varied results. The limited pre-clinical and clinical population sizes have hampered the success of these trials. To understand the microbiome associated with ASD, we employed whole metagenomic shotgun sequencing to classify microbial composition and genetic functional potential. Despite being one of the most extensive ASD post-synbiotic assessment studies, the results highlight the complexity of performing such a case–control supplementation study in this population and the potential for a future therapeutic approach in ASD.

## INTRODUCTION

Autism spectrum disorder (ASD) is a complex neurodevelopmental condition characterized by significant impairments in social interaction and communication, repetitive and restricted behaviors, and deficits in sensory reactivity (1). Although there are wide variations in the severity of symptoms among ASD patients, gastrointestinal (GI) symptoms are a common comorbidity. The percentage of patients with ASD presenting GI issues varies from study to study. Still, there is a general trend that ASD patients often suffer from diarrhea, constipation, bloating, gastroesophageal reflux, and/or abdominal pain (2–4). The high frequency of GI problems in ASD patients raised a question about the relationship between autism-related symptoms and the gut microbiome (5). Many studies have accumulated evidence that dysbiosis might affect behavioral and gastrointestinal symptoms in children with ASD (6–13). The bidirectional relationship between the gut and the brain, known as the gut–brain axis, is a widely accepted concept (14).

Although most studies conclude that patients with ASD have a distinctive gut microbiome, there is inconsistency in the taxa reported between these studies (15). Some studies show little to no differences between patients with ASD and neurotypical cohorts (16–19), while others show significant differences between these cohorts (4, 12, 20, 21). This inconsistency is partially explained by the complexity of ASD, given the heterogeneity of symptoms and their severity (22, 23) and the heterogeneity of their microbiome. However, methodological differences in sample and data collection, a lack of standardization in GI symptom diagnosis, small cohorts, and differences in diet, geography, socioeconomics, and ethnicity all compound efforts to create universal feature predictions for this complex disorder (10, 22–25). For example, a recent non-invasive study analyzed the diet, fecal microbiome, and fecal consistency of 247 children, where 99 children were diagnosed with ASD (19), and found that dietary differences driven by food preferences in children with autism may be the primary driver for observed differences in the microbiome, calling into question whether the microbiome has any causal influence on ASD (25).

In contrast, microbiota transplant therapy (a form of fecal transplant) to influence the gut microbiome of children with ASD showed efficacy in altering ASD symptoms in the long term (3, 26, 27). Our interpretation is that the ASD microbiome is very heterogeneous. Unlike *Clostridioides difficile* infections that involve a single pathogen, ASD likely involves multiple pathogens, the lack of numerous beneficial bacteria, or both with different pathogens and different losses of beneficial bacteria.

Recently, a significant focus has been on developing precision intervention studies to improve responsiveness to personalized treatments (28, 29). The variability in the gut microbiome of people with ASD may indicate that precision supplementation would be needed to see a more robust improvement in symptoms. Here, we present a study to examine whether a commercial personalized synbiotic formulation designed based on the baseline metagenomic analysis of each person’s stool can influence ASD symptoms and microbiome characteristics. We developed customized synbiotics for individuals based on microbial composition using metagenomic analysis from fecal samples and a personal health and diet survey of each person. This synbiotic is a personalized combination of typically one to two prebiotics and four to eight probiotics chosen from a list of more than 100 scientifically studied possible ingredients to modulate the individual’s gut microbiome. We performed whole-genome shotgun metagenomics on the gut microbiomes of 296 children and adults with ASD vs 123 neurotypical controls at the baseline and following 3 months of customized synbiotic consumption for a subset of the ASD group and investigated whether the supplement influenced ASD and GI symptoms.

## MATERIALS AND METHODS

### Participants and sample collection

Sun Genomics partnered with Arizona State University’s Autism (ASU)/Asperger’s Research Program and the Biodesign Center for Health Through Microbiomes to collaborate on this Institutional Review Board (IRB) study. Sun Genomics is a microbiome health company dedicated to crafting customized precision probiotics formulas for its customers’ unique gut health needs using the possibilities of whole-genome sequencing. The study collaboration with ASU enabled the investigation of the potential connection between the gut microbiome and autism spectrum disorder using the Sun Genomics product Floré (research edition).

Participants were invited to participate in this study if they were new customers of Sun Genomics and had purchased Floré (https://flore.com/pages/flore-research-edition) but had not yet started synbiotic supplementation. They viewed a study ad and a written consent form explaining the study and their potential role and were invited to participate. The consent form was signed by the parent of the person with ASD or the adult with ASD if they did not have a legal guardian. The study was approved by the IRB of Arizona State University as STUDY00012299 and registered on clinicaltrials.gov before enrolling participants.

The inclusion criteria for the ASD group included the following:

1. A new client of Sun Genomics (has applied for testing and supplementation but has not yet begun supplements).
2. Diagnosis of ASD (documentation of a clinical diagnosis of ASD) was obtained from participants and then verified by a parental or self (19 or older) evaluation of the social responsiveness scale (SRS2) with a raw score above 60.
3. Children and adults aged 2.5–75 years.

The exclusion criteria included the following:

1. Antibiotic use in the last 2 months (not counting topical antibiotics)
2. Any changes in medications, nutritional supplements, or therapies in the last 2 months or plans to change them during the first 3 months of synbiotic supplementation.

A total of 296 participants with ASD were included in this study (Table 1). The ASD group was matched with 123 neurotypical, age-matched controls from the Sun Genomics customer base to compare the whole-genome sequencing of their fecal sample. Based on their health and diet survey, the controls were further screened for having no neurodisabilities, serious gastrointestinal disorders, or disease.

**TABLE 1:** Study cohort demographics^*a*^

		ASD	NT
*N*		296	123
Age		10.41 (7.14)	10.74 (8.71)
% male		79.7%	52.0%
Shannon index		3.90 (0.48)^*^	4.04 (0.38)^*^
PGIA	Overall	2.81 (0.87)	–
SCARED	Overall	19.34 (15.46), 26.11%	–
	Panic disorder	19.34 (15.46), 17.83%	–
	Generalized anxiety disorder	4.04 (4.44), 19.11%	–
	Separation anxiety	4.42 (3.74), 42.04%	–
	Social anxiety disorder	5.49 (4.12), 29.30%	–
	Significant school avoidance	1.52 (1.87), 27.39%	–
SRS2	Overall	80.08 (10.36)	–
	Mild to moderate	6%	–
	Moderate	29%	–
	Severe	6%	–
GSRS	Overall	2.25 (0.97)	–
	Abdominal pain	3.37 (1.26)	–
	Constipation	2.69 (1.61)	–
	Diarrhea	2.01 (1.22)	–
	Ingestion syndrome	2.39 (1.8)	–
	Reflux syndrome	1.69 (1.16)	–
Nutrition	Overall	21.07 (14.71)	–
	Excellent/very good	17.5%	–
	Average	22.6%	–
	Below average/poor	59.8%	–

^*a*^ Listed are the number of subjects, age, and gender of each cohort and the summaries of surveys taken by ASD study participants. The PGIA survey is the parental global impressions of autism. SCARED is the screen for childhood-related anxiety disorders. SRS2 is the social responsiveness scale. GSRS is the gastrointestinal symptom response scale. The nutrition survey assesses dietary habits and the number of daily servings of various food categories. Values within the parentheses indicate SD from the mean. Percentages indicate the population proportion that falls within a subcategory (SCARED) or indicate a specific condition or phenotype (SRS2 and nutritional assessment surveys).

The ASD group completed extensive baseline evaluations regarding dietary and nutritional habits, behavior, birth and infancy history, allergies, social responsiveness, anxiety-related disorders, and gastrointestinal symptoms. The surveys collected were (i) parental global impressions of autism (PGIA), (ii) screen for childhood-related anxiety disorders (SCARED), (iii) social responsiveness scale (SRS2), (iv) gastrointestinal symptom rating scale (GSRS), and (v) nutritional assessment. The PGIA questionnaire assesses observable behaviors in children with ASD, including language, cognition, play skills, sociability, and hyperactivity. On average, the cohort had moderate severity of symptoms (Table 1). The SCARED survey assesses five classes of anxiety, including panic disorder, generalized anxiety disorder, separation anxiety, social anxiety disorder, and significant school avoidance. Each of the categories has a scoring threshold that may suggest the presence of an anxiety disorder. Not all surveys were required for participation or as inclusion criteria for the study. The ASD study surveys were not collected from the control cohort, as these participants were collected at different times for different purposes.

The nutritional survey asks for the number of servings of different food types consumed daily and everyday dietary habits. Each question has a positive or negative value depending on the kind of food assessed. For example, servings of nuts and seeds have a positive value, while refined grains have a negative score based on daily consumption. Other questions consider whether the diet includes artificial colors, flavors, or additives and whether the diet is organic. Diet quality was determined by summing up the score of the survey. Higher scores are associated with excellent or good diet quality, and lower scores are associated with poorer diets. Diet from the control cohort was assessed using the health and diet survey.

Fecal samples were collected via the Floré research edition collection components for metagenomic sequencing. The fecal sample was organized by the parent or participant of the study with provided component instructions. Fecal samples from the control population were collected with the gut microbiome collection box (Floré gut health test). Samples are collected in a proprietary stabilization buffer and on a swab and shipped for 2-day delivery at ambient temperature. A proprietary minimal media was used for the swab collection. To ensure the efficiency of the buffer, a known positive control was included in every batch. The buffer was internally validated to confirm a similar result compared to a fresh stool sample. Upon receipt at Floré Laboratories, samples are refrigerated at 4°C and processed within 3 days.

As part of the longitudinal study, an additional timepoint from the ASD population was collected after 3 months of the supplementation. Of the ASD cohort, there were 170 participants with a second timepoint. Different for each participant, formulations included four to eight probiotic strains and one to two prebiotics at different concentrations. The list of formulations is included in Table S1. Participants were instructed to take it daily. The precision synbiotics were formulated based on the gut microbiome taxonomic profiles and health and diet surveys. Probiotic strains were selected based on the following: (i) The most recent scientific literature in clinical research studies is curated, preferably on human subjects from about 10,000 papers for scientific merit. Other relevant basic research studies focusing on species characterization are also considered, specifically focusing on probiotic modulation. This includes mechanistic studies done on cell lines if they are aimed at understanding or improving human gut microbiome health or disease. (ii) Physiological concerns listed on the health and diet survey are also considered.

The decision metrics for synbiotic combinations consist of several steps, primarily including choosing probiotic species studied for their ability to modulate the microbiota–gut–brain axis and improve ASD-specific characteristics. Flore’s health and diet survey completed by each participant helps us address their specific symptoms, including ASD-associated social, behavioral, and GI concerns. For example, several probiotics have been shown to relieve gastrointestinal symptoms such as constipation (30–32), bloating, gassiness (33), and diarrhea (34, 35). The health and diet surveys also guided which prebiotics to avoid based on specific allergies or sensitivities. Species and strains are carefully picked, based on our clinical literature curation database, to help out-compete any pathogens or reduce their overgrowth. Floré’s proprietary formulation database, which includes extensive information on the success rates of our formulation inventory, is used to validate the final product. Calculation models and decision matrix analysis help determine the type, dose, and cocktail of microbes to include in the formula (patent 10428370B2). On average, there was a 5–6-month difference between the generation of the first baseline timepoint and the second timepoint sample receipt and processing. After the second fecal sample collection, GSRS, PGIA, SCARED, and SRS2 follow-up surveys were collected again.

### Metagenomic sequencing and bioinformatic analysis

DNA extraction and library preparation methods are previously described in reference 36. Briefly, DNA was extracted and purified using a proprietary process (patents 10428370 and 10837046). Library prep and size selection were performed with NEBNext reagents and MagJet magnetic beads, respectively. Library quantitation was performed with qPCR before normalization. All reagents used in the DNA extraction and library prep processes were labeled sterile and DNase-free. Libraries were sequenced on an Illumina NextSeq 550 using 150 bp × 150 bp paired-end reads (Illumina, San Diego, CA). Once sequenced, reads were quality-filtered and processed to remove human reads. The mean sequencing depth was 17.15M total reads for the ASD cohort and 6.14M reads for the control cohort. The mean classified reads were 71% for both cohorts. For taxonomic analysis, reads were aligned to a hand-curated database of 23,000 microbial species. Our proprietary bioinformatics pipeline includes quality filters before taxonomy assignment. Quality trimming of raw sequencing files may include removing sequencing adaptors or indexes; trimming 3′ or 5′ end of reads based on quality scores (Q20>); and removing reads based on quality scores, GC content, or non-aligned basepairs. Alignment of processed sequencing files was done using a custom microbial genome database consisting of sequences from Refse, Greengeens, HMP, NCBI, PATRIC, or other public/private data repositories and may include other in-house data sets. This database may be used as a full genome alignment scaffold, k-mer fragment alignment, or other schemes practiced in metagenomics and bioinformatics. Metabolic pathways and gene families were determined using HUMAnN2 with the MetaCyc and UniRef90 databases (37).

### Statistical analyses

The downstream statistical analyses in this paper were performed in R. Permutational multivariate analysis of variance (PERMANOVA) has the “adonis” function from the “vegan” package (38). Bray–Curtis dissimilarity was calculated with the “vegan” package and was used for principal coordinate analysis (38). Pearson correlations were used to investigate parametric relationships between variables of interest. Random forest analysis was performed to identify variables of importance between the two cohorts in the microbiome, pathways, and gene family data sets (39, 40). Subsequent significance testing with false discovery rate (FDR) corrections was performed on the top 50 random forest variables of importance to compare the relative proportions of microbes from each cohort. The Mann–Whitney *U* test or Wilcoxon rank sum tests were performed to determine statistical significance between observations. FDR corrections were used to control for multiple hypothesis comparisons. The taxonomic composition data were rarefied, and the sample sum was normalized to account for differences in sequencing depth. The pathway and gene family data sets were sample sum normalized to account for differences in sequencing depth. Stochastic gradient boosting models were performed with the caret package to build a predictive model of microbiome features associated with ASD (41). For the model, there was 75%/25% train and test split of the data. We tested an interaction depth of 1, 5, and 9 for model optimization. The number of trees for optimization started at 50 up to 1,500, stepwise by 50 with 0.1 shrinkage. The minimum number of training set samples in a node was set to 20. When training, samples were down-sampled to match the smaller number of the control samples in the training set. We used the train control method “repeatedcv” with five repeats and a twoClassSummary function. As for training the model, the method used was “gbm” with an “ROC” metric with the above parameters. The optimal results from training optimization were a shrinkage of 0.1, interaction depth of 9, and 1,350 trees with an ROC of 0.95 and sensitivity of 0.90.

### STORM

The STORMS version 1.03 checklist for this study is available at https://doi.org/10.5281/zenodo.8215858.

## RESULTS

### Subject demographics and behavioral, gastrointestinal, and nutritional survey summaries

A total of 296 study participants with ASD and 123 neurotypical subjects were included in this study (Table 1). All subjects in the ASD cohort obtained and submitted clinical diagnosis documents for participation in the study. ASD and control cohorts were age-matched, with mean ages of 10.41 and 10.74, respectively. The ASD cohort was 79.7% male, while the control was 52.0% male (Table 1). Of the ASD cohort, 89% was from the United States, 3.8% was from Canada, 6% was from Europe, and less than 1% was from Australia or Israel. An initial analysis found no significant differences in microbial alpha or beta diversity between sexes in either cohort, so the control group was not restricted to match the gender of the ASD group. First, we calculated alpha diversity on rarefied microbiome data. To test for differences, we performed a Wilcoxon rank sum test for the alpha diversity between male and female profiles for each of the ASD and control cohorts. We did not find a significant difference in either cohort (*P*-value >0.05). Of the control cohort, 96% was from the United States; 3.5% was from Europe, Canada, or Australia; and less than 1% was from Singapore.

Of the 296 participants, 126 of the subjects dropped out of the study after consenting. Parents of children with ASD or adult customers with ASD either filed complaints directly or decided to drop out of the study. Not all participants provided a reason for dropping out of the study, but the two primary reasons for dropping out were price (21%) and a lack of perceived benefit (34%). Three complaints were received during the study related to customer service, and the third regarded a lack of perceived benefit from the supplementation.

Using the information collected from the surveys, 26% of the ASD population may have an anxiety disorder, as assessed by the SCARED survey (Table 1). As for each specific anxiety disorder, including panic disorder, generalized anxiety disorder, separation anxiety disorder, social anxiety disorder, and school avoidance, 17%–42% of the study participants may have at least one of the anxiety disorders (Table 1). The SRS2 survey indicates that 64% of the ASD cohort has severe difficulties in social responsiveness (Table 1). A smaller proportion of the cohort has mild to moderate and moderate difficulties, with 6% and 29%, respectively (Table 1).

From a rating scale of no discomfort to very severe discomfort, the ASD cohort, on average, had slight to mild discomfort as assessed with the GSRS survey, which evaluates abdominal pain, reflux syndrome, diarrhea syndrome, indigestion syndrome, and constipation (Table 1). Abdominal pain had a higher average subtype score with mild to moderate discomfort, while reflux syndrome was lower with no to slight discomfort (Table 1). The nutritional survey found that approximately 60% of the ASD cohort had below-average or poor diets, 22.6% had average, and 17.5% had excellent or very good diets (Table 1). Higher scores calculated from the nutritional survey are associated with good diets, and lower scores are associated with poor diets (also explained in Materials and Methods). In a broad comparison of diets of children and adolescents in the United States as measured by the National Health and Nutrition Examination Study, 36.3% consumed fast food within a given day, and 75.3% consumed fruit on a given day, which decreased with age. There may be a broad trend of dietary habits from healthy to poor diets in children and adolescents in the United States. Of the dietary preferences, approximately a third of the ASD participants practiced casein-free, gluten-free, or combined gluten- and casein-free diets, and 5% were vegetarian. Nearly 65% took additional nutritional supplements.

### Microbial differences in the ASD cohort were based on phenotypic groupings

**Fig 1:**
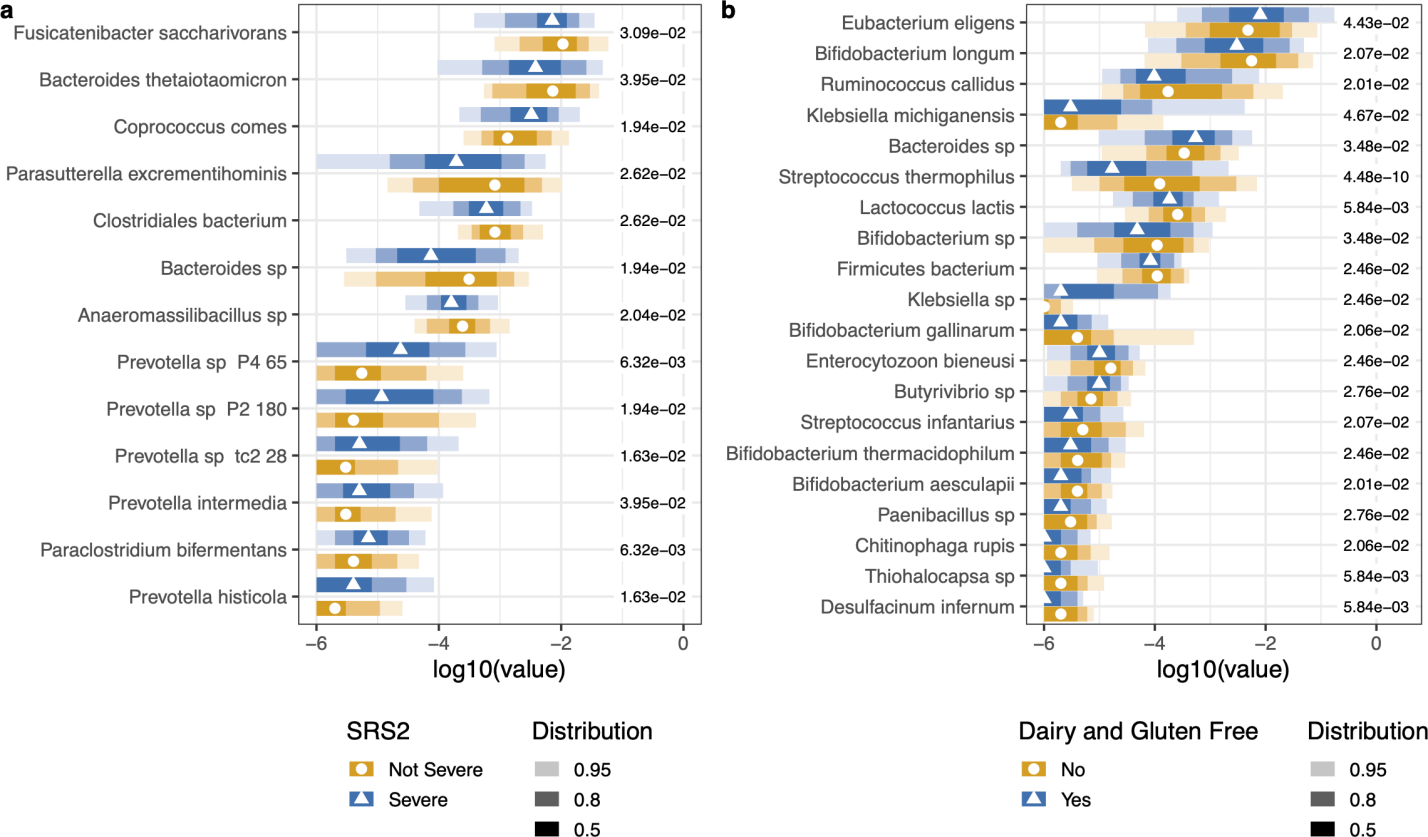
Microbes associated with ASD subpopulations. The top 50 microbes were selected from the variables of importance from a random forest model. (a) Microbes significantly differed between subjects with severe or non-severe scores from the SRS2 survey. (b) Microbes significantly differed between subjects with dairy-, gluten-, or dairy and gluten-free diets versus those who consumed gluten and dairy. Kruskal–Wallis with *post hoc* Dunn tests were performed. The points represent the median value. The shading of color along the bars indicates the distribution of the data across the *x*-axis. Of the data points, 95% of the data fall within the lightest shade, 80% of the data fall within the medium shade, and 50% of the data fall within the darkest shaded bar. The different colors represent the two cohorts. The value to the right of the bars is the adjusted *P*-value for each test.

Within the ASD cohort, a spectrum of subjects differ in anxiety, difficulties in social responsiveness, and nutritional habits. To investigate whether microbiome differences exist in these subpopulations, we performed random forests on classification bins to identify microbial features that differentiate phenotypic differences in the ASD cohort. From the SCARED survey of the 26% of ASD participants that may have an anxiety disorder versus participants that did not, no significant microbial species differentiated the cohorts based on anxiety. Of the 64% of the participants who had severe difficulties in social responsiveness versus those with mild to moderate difficulties based on the SRS2 survey, there were significant differences in the proportion of specific taxa between participants with mild and severe social responsiveness, including *Prevotella* spp., *Bacteroides,* and *Fusicatenibacter* (Fig. 1). In nutrition, while there were no significant differences in microbes based on whether participants had either good or poor diets, there were significant differences in participants who had a gluten and dairy-free diet versus participants who do consume dairy and gluten (Fig. 1). Probiotic species, *Bifidobacterium* spp., *Lactococcus lactis,* and *Streptococcus* spp., were significantly higher in proportion in subjects with dairy and gluten-inclusive diets (Fig. 1).

### Microbial diversity differed in the ASD cohort relative to controls and improved after synbiotic supplementation

**Fig 2:**
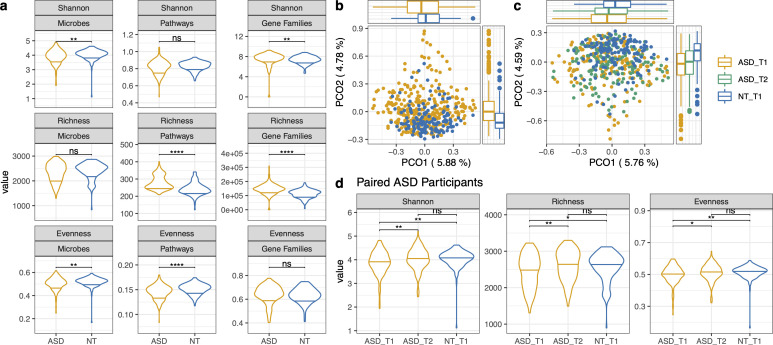
Diversity comparisons between ASD and neurotypical cohorts and paired longitudinal ASD subjects. (a) Shannon index, richness, and evenness across microbiome composition, metagenomic pathways, and gene families between cohorts. (b) PCO of the ASD and neurotypical cohorts. Yellow dots represent ASD, and blue dots represent NT. The boxplots along each axis represent the distribution points within the PCO. The Wilcoxon test for comparisons between cohorts resulted in a *P*-value =0.0102 for PCO1 and a *P*-value = 4.68*e*−12 for PCO2. (c) PCO of paired ASD timepoints 1 and 2 and neurotypical timepoint 1 samples. For PCO1, ASD_T1 vs NT_T1 *P*.adj = 0.006, ASD_T2 vs NT_T1 *P*.adj = 0.0165. For PCO2, ASD_T1 vs NT_T1 *P*.adj = 2.43*e*−9, ASD_T1 vs NT_T1 *P*.adj = 2.62*e*−8. Principal coordinates were calculated based on a Bray–Curtis dissimilarity matrix. (d) Alpha diversity at baseline (timepoint 1) for ASD and neurotypical and subsequent timepoint 2 for the ASD cohort. Wilcoxon rank sum tests were performed for all tests with Benjamini–Hochberg *post hoc* corrections for multiple comparisons. ^*^P-value < 0.05; ^**^P-value < 0.01; ^****^P-value < 0.0001; ns, not significant.

Alpha and beta diversity metrics were calculated to compare microbial diversity between the ASD and control cohorts. At baseline, the ASD cohort had a significantly lower Shannon score and microbial evenness than the control cohort (Fig. 2a). There was no significant difference in beta diversity as calculated by a PERMANOVA based on a Bray–Curtis dissimilarity matrix (*P*-value = 0.065 and *R*^2^ = 0.00319). The microbiome beta diversity was also displayed through principal coordinate analysis. Significant differences existed in the PCO1 and PCO2 coordinates of the ASD and NT baseline cohorts (Wilcoxon test, *P*-value = 0.0102 and 4.68*e*−12, respectively, Fig. 2).

The analysis included 170 ASD participants with data for baseline and after 3 months of supplementation with Floré. In the paired samples, there was a significant increase in the three assessed alpha diversity indexes (Shannon, richness, and evenness) at timepoint 2 relative to timepoint 1 at baseline (Fig. 2). The microbial alpha diversity of ASD at timepoint 2 was no longer significantly different compared to the neurotypical cohort (Fig. 2d). There was a significant difference between the PCO1 and PCO2 axes between both timepoints of ASD and the NT baseline (Wilcoxon test, *P*.adj <0.05, Fig. 2). There was no significant difference in principal coordinates between ASD timepoints 1 and 2 (Fig. 2b and c).

### Species evenness and richness correlated negatively with anxiety and positively with daily servings of fruit

To investigate whether differences in microbial alpha diversity in the ASD cohort were related to any of the observed behaviors, nutrition, gastrointestinal discomfort, social responsiveness, or anxiety, Pearson correlations were calculated to find significant trends. Microbial evenness was inversely correlated to the SCARED total score, such that lower evenness was associated with higher anxiety (Table 2; Fig. S1). Alpha diversity was not correlated with the nutritional assessment, PGIA, SRS2, or GSRS surveys at baseline. Of the food groups assessed by the nutritional assessment survey, microbial richness was significantly positively correlated to the number of daily fruit servings (Table 2; Fig. S1). The nutritional assessment survey was inversely correlated to the PGIA scores (Table 2; Fig. S1). The PGIA score assesses observable behaviors, such as language, cognition, play skills, sociability, and hyperactivity, where a higher score aligns with more severe symptoms. This result indicates that individuals with lower (worse) nutrition had higher (worse) ASD severity.

**TABLE 2:** Pearson correlations between total survey scores and alpha diversity measurements^*a*^

Variable 1	Variable 2	*R*	*P*-value
Nutritional assessment	Shannon	0.037	0.69
	Evenness	0.015	0.87
	Richness	0.13	0.15
	**PGIA**	**−0.19**	**0.025**
Nutrition—fruit	Shannon	0.087	0.35
	Evenness	0.063	0.5
	**Richness**	**0.2**	**0.031**
PGIA	Shannon	0.014	0.83
	Evenness	0.0096	0.89
	Richness	0.037	0.58
SRS2	Shannon	0.046	0.51
	Evenness	0.039	0.58
	Richness	0.069	0.33
GSRS	Shannon	−0.052	0.45
	Evenness	−0.046	0.5
	Richness	−0.076	0.27
SCARED	Shannon	−0.17	0.051
	**Evenness**	**−0.17**	**0.045**
	Richness	−0.097	0.26

^*a*^ *R* and *P*-values were calculated for each comparison between surveys taken by the ASD population and the microbiome diversity Shannon index, evenness, and richness. Significant correlations between different survey scores and alpha diversity indices are bolded.

### Taxonomic, metabolic pathway, and gene family proportions differed significantly between ASD and neurotypical cohorts

**Fig 3:**
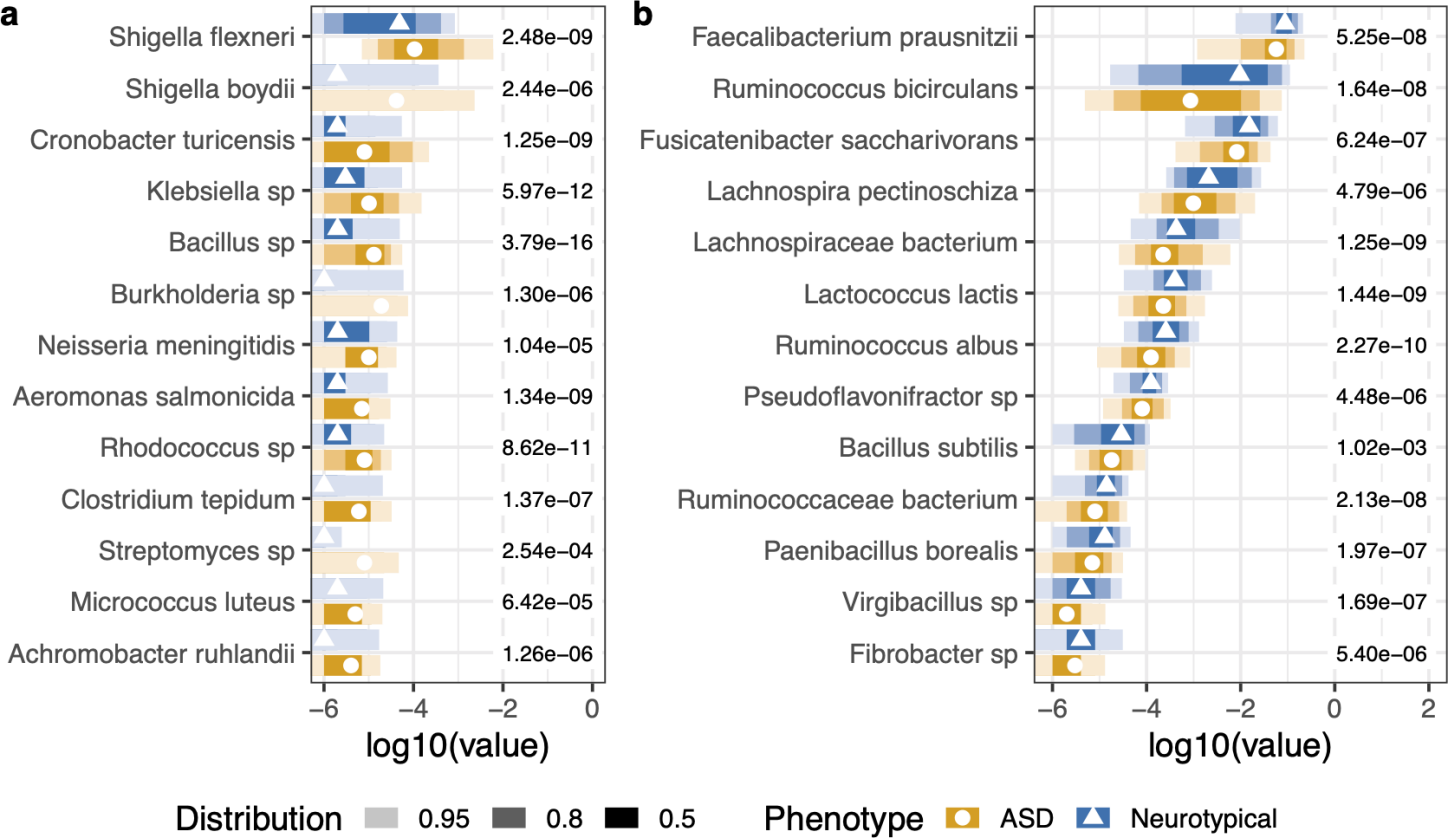
The differential proportion of microbes between ASD and NT cohorts at baseline. The top 50 microbes were selected from the variables of importance from a random forest model. Microbes listed in (a) were detected at higher proportions in the ASD cohort, while microbes in (b) were detected at lower proportions in the ASD cohort relative to the neurotypical cohort. Wilcoxon rank sum tests with Benjamini–Hochberg *post hoc* corrections for multiple comparisons were used to determine a significant differential proportion of microbes between ASD and NT cohorts. The points represent the median value. The shading of color along the bars indicates the distribution of the data across the *x*-axis. Of the data points, 95% of the data fall within the lightest shade, 80% of the data fall within the medium shade, and 50% of the data fall within the darkest shaded bar. The different colors represent the two cohorts. The value to the right of the bars is the adjusted *P*-value for each test.

Microbial taxonomic, metabolic pathway, and gene family features significantly differed between the ASD and control cohorts. The top 50 features of importance were determined by random forest analysis. From the random forest model, the out-of-the-box error rate estimate was 17.54%. With additional significance testing with corrections for multiple hypothesis testing of the top 50 variables, we identified 28 significant microbial features associated with the ASD or control cohorts (Fig. 3). *Achromobacter, Aeromonas, Bacillus* sp.*, Burkholderia, Clostridium, Cronobacter, Klebsiella, Micrococcus, Rhodococcus, Shigella,* and *Streptomyces* had significantly greater proportions in the ASD cohort, which includes some species with pathogenic potential (Fig. 3a). Meanwhile, *Bacillus subtilis, Faecalibacterium, Fibrobacter, Fusicatenibacter, Lachnosphira, Lactococcus, Paenibacillus, Pseudoflavonifractor, Ruminococcus,* and *Virgibacillus* were all at a larger proportion in the microbiome of controls, some of which are potentially beneficial (Fig. 3b). Training a stochastic gradient boosting model based on taxonomy of sampled neurotypical and ASD populations, the AUC of the ROC curve was 0.945 with tuning optimizations. However, the validation data set had an accuracy of 21.8%, suggesting a low level of predictive accuracy.

**Fig 4:**
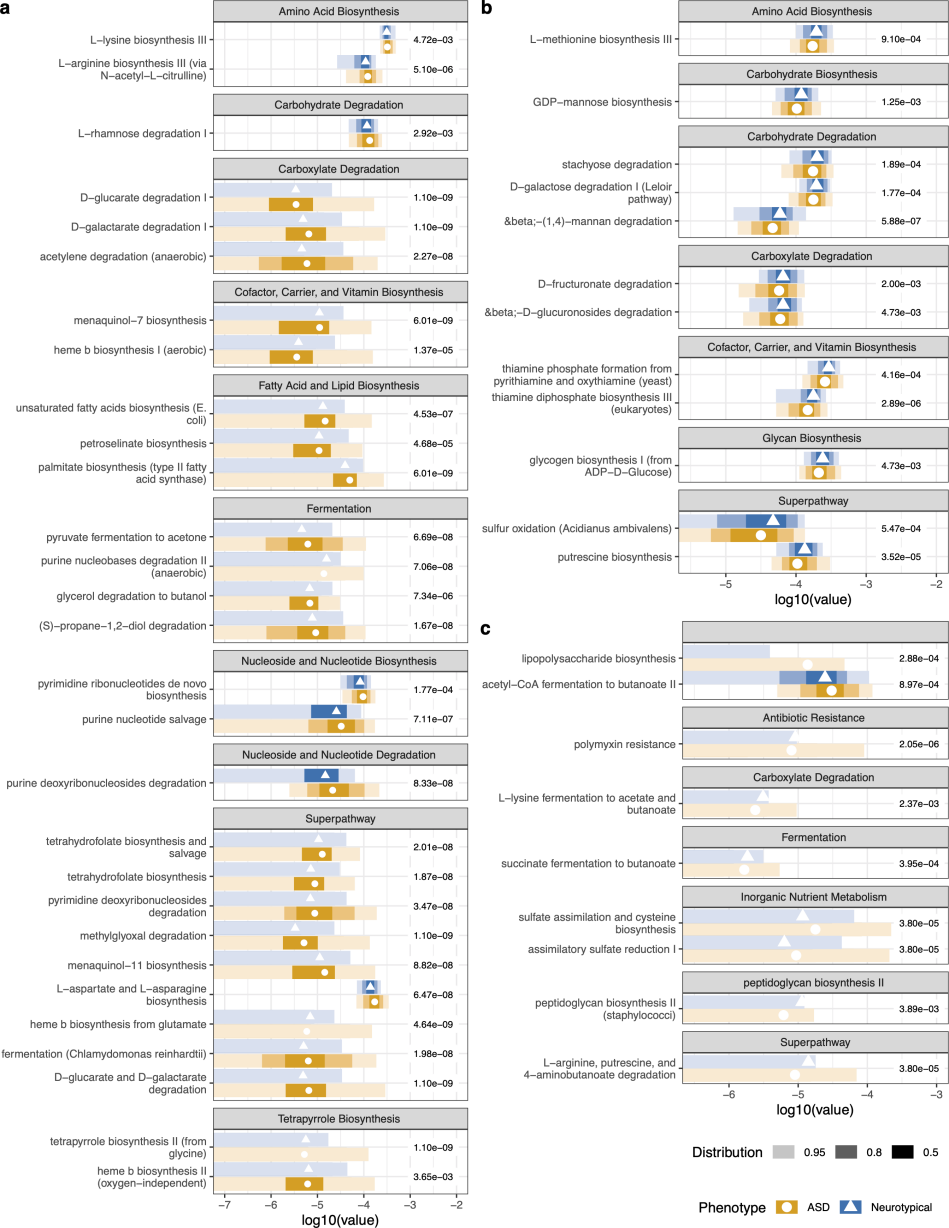
The differential proportion of pathways between ASD and NT cohorts at baseline. The top 50 pathways were selected from the variables of importance from a random forest model. Pathways listed in (a) were detected at higher proportions in the ASD cohort, while pathways in (b) were detected at lower proportions in the ASD cohort relative to the neurotypical cohort. (c) Pathways assessed with *a priori* knowledge in current ASD literature. Wilcoxon rank sum tests with Benjamini–Hochberg *post hoc* corrections for multiple comparisons were used to determine a significant differential proportion of pathways between ASD and NT cohorts. The points represent the median value. The shading of color along the bars indicates the distribution of the data across the *x*-axis. Of the data points, 95% of the data fall within the lightest shade, 80% of the data fall within the medium shade, and 50% of the data fall within the darkest shaded bar. The different colors represent the two cohorts. The value to the right of the bars is the adjusted *P*-value for each test.

The MetaCyc Metabolic Pathway Database was used to annotate the functional pathway potential of each metagenome. Using random forest to identify functional features that differentiated between cohorts, we demonstrated that ASD participants had a significantly greater proportion of metabolic pathways associated with biosynthesis of amino acids; cofactor, carrier, and vitamins; fatty acid and lipids; nucleotides; tetrapyrrole; degradation of carboxylates; and fermentation of pyruvate and alcohols and fermentation to short-chain fatty acids when compared to controls (Fig. 4a). The out-of-the-box estimate of error rate for the pathway potential was 23.45%. Pathways that were significantly lower in proportion in ASD metagenomes included carbohydrate degradation, sulfur oxidation, and thiamine biosynthesis (Fig. 4b). Additional pathways that have previously been identified as associating with ASD symptoms, including lipopolysaccharide (LPS) biosynthesis (42), antibiotic resistance (43), sulfur compound metabolism (44), and gamma-aminobutyric acid (20), were also significantly different in ASD compared to control cohorts (Wilcoxon rank sum tests with Benjamini–Hochberg *post hoc* corrections for multiple comparisons; Fig. 4c).

**Fig 5:**
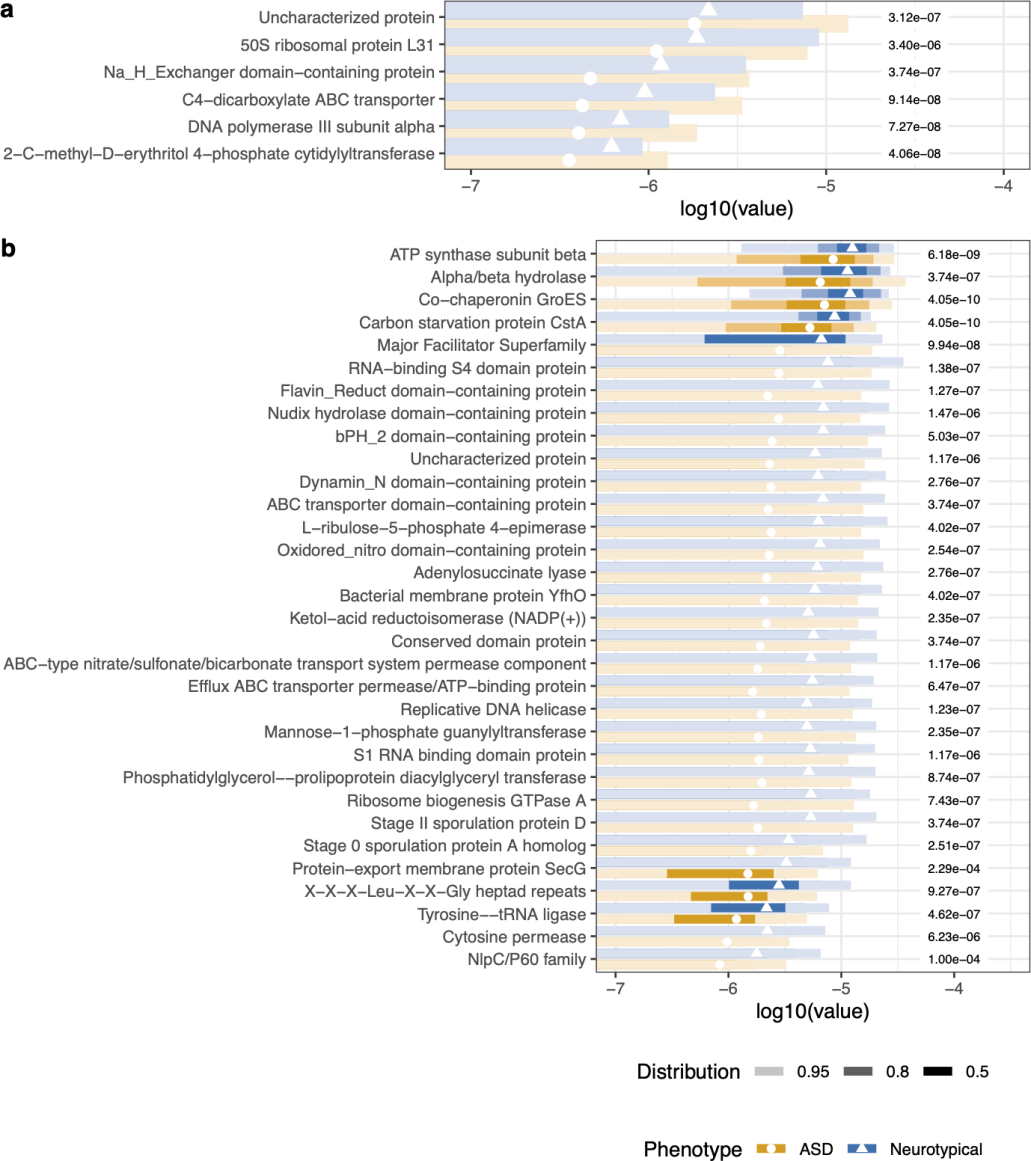
Differential proportions of gene families between ASD and NT cohorts at baseline. The top 50 gene families were selected from the variables of importance from a random forest model. Gene families listed in panel a were detected at higher proportions in the ASD cohort, while gene families in panel b were detected at lower proportions in the ASD cohort relative to the neurotypical cohort. Wilcoxon rank sum tests with Benjamini–Hochberg *post hoc* corrections for multiple comparisons were used to determine a significant differential proportion of gene families between the ASD and NT cohorts. The points represent the median value. The shading of color along the bars indicates the distribution of the data across the *x*-axis. Of the data points, 95% of the data fall within the lightest shade, 80% of the data fall within the medium shade, and 50% of the data fall within the darkest shaded bar. The different colors represent the two cohorts. The value to the right of the bars is the adjusted *P*-value for each test.

The UniRef (UniRef90 201901b) database was the reference database for the gene families detected in the metagenomic data. To reduce the computational burden for the statistical analyses of the gene family data set, the sum threshold was >1,000 reads per kilobase per gene family. Random forest was used to identify the main features of importance. The out-of-the-box estimate of the error rate for gene families was 15.35%. Out of the top 50 gene families from the variables of importance of a random forest model, six gene families were at a significantly greater proportion, and 44 were at a significantly lower proportion based on mean values in the ASD cohort (Wilcoxon rank sum tests with Benjamini–Hochberg *post hoc* corrections for multiple comparisons; Fig. 5a and b). Most gene families lower in ASD were annotated from *Ruminococcus* spp., *Fusicatenibacter saccharivorans,* and *Faecalibacterium prausnitzii*, which reflects the lower proportion of those microbes detected in the ASD cohort relative to the controls (Fig. 3b).

**Fig 6:**
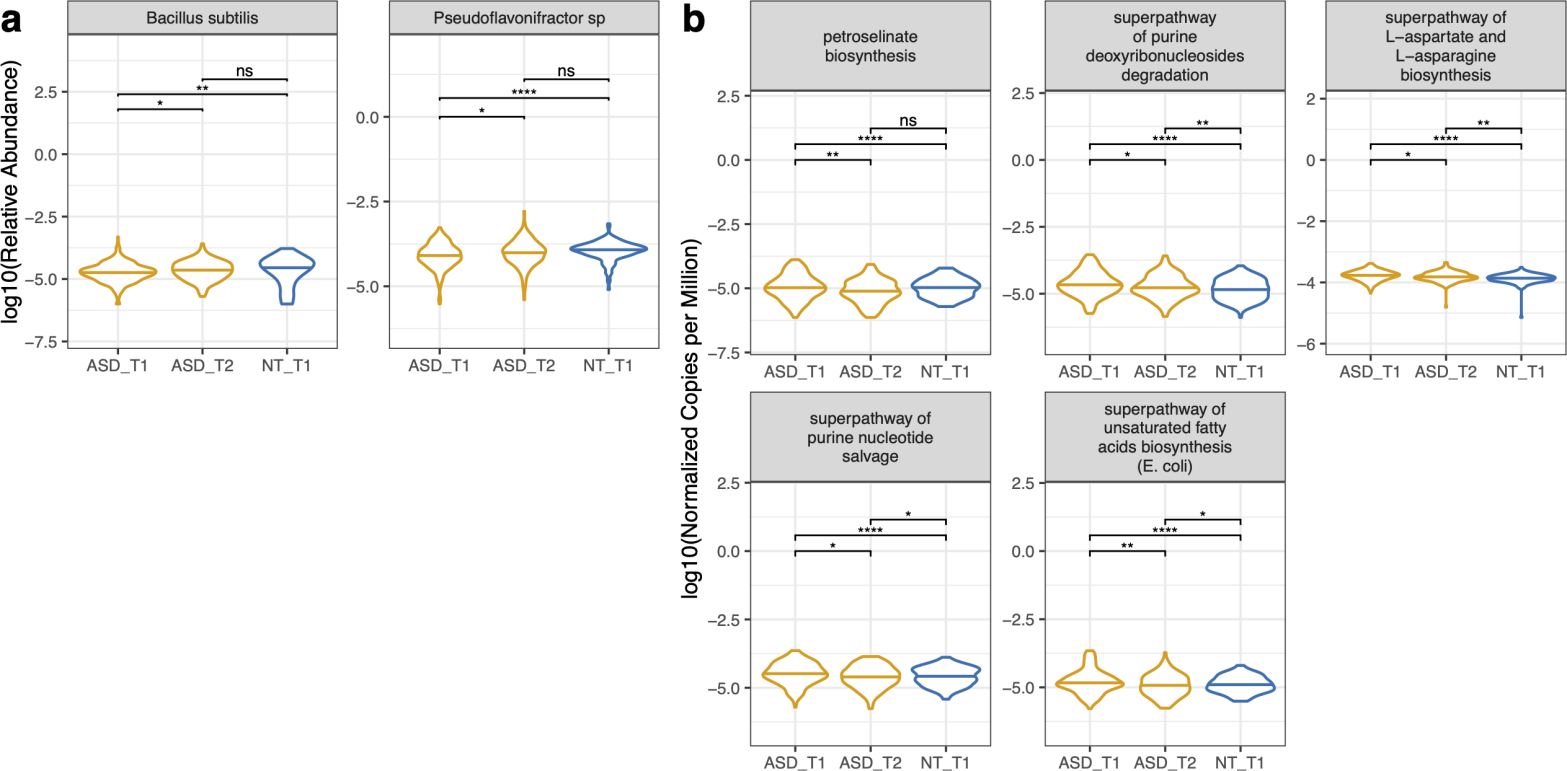
Longitudinal microbiome trends. Longitudinal assessments were made from the top 50 variables of importance obtained from the baseline comparisons of the microbiome composition, pathway potential, and gene family data sets. ASD-paired samples, where subjects had both timepoints, were compared to the baseline neurotypical cohort. (a) Microbes and (b) metagenomic pathways listed were significantly different between baseline timepoint 1 and timepoint 2 in the ASD cohort. Wilcoxon tests with Benjamini–Hochberg *post hoc* corrections were calculated. ^*^P-value < 0.05; ^**^P-value < 0.01; ^****^P-value < 0.0001; ns, not significant.

There were several microbes and pathways that were significantly different in proportion after synbiotic supplementation compared to baseline for the 170 ASD participants that completed supplementation (Fig. 6). *Bacillus subtilis* and *Pseudoflavonifractor* sp. *An85* increased in proportion post-synbiotic supplementation and became not significantly different from their proportion in the neurotypical cohort (Fig. 6a). The proportion of the metabolic pathway responsible for petroselinate biosynthesis decreased post-supplementation and became not significantly different compared to their proportion in the neurotypical cohort (Fig. 6b). Meanwhile, L-aspartate and L-asparagine biosynthesis, unsaturated fatty acid biosynthesis, purine deoxyribonucleoside degradation, and purine nucleotide salvage all significantly decreased post-synbiotic and became more like controls (Fig. 6b).

### Behavioral and gastrointestinal symptom improvement with follow-up surveys

**Fig 7:**
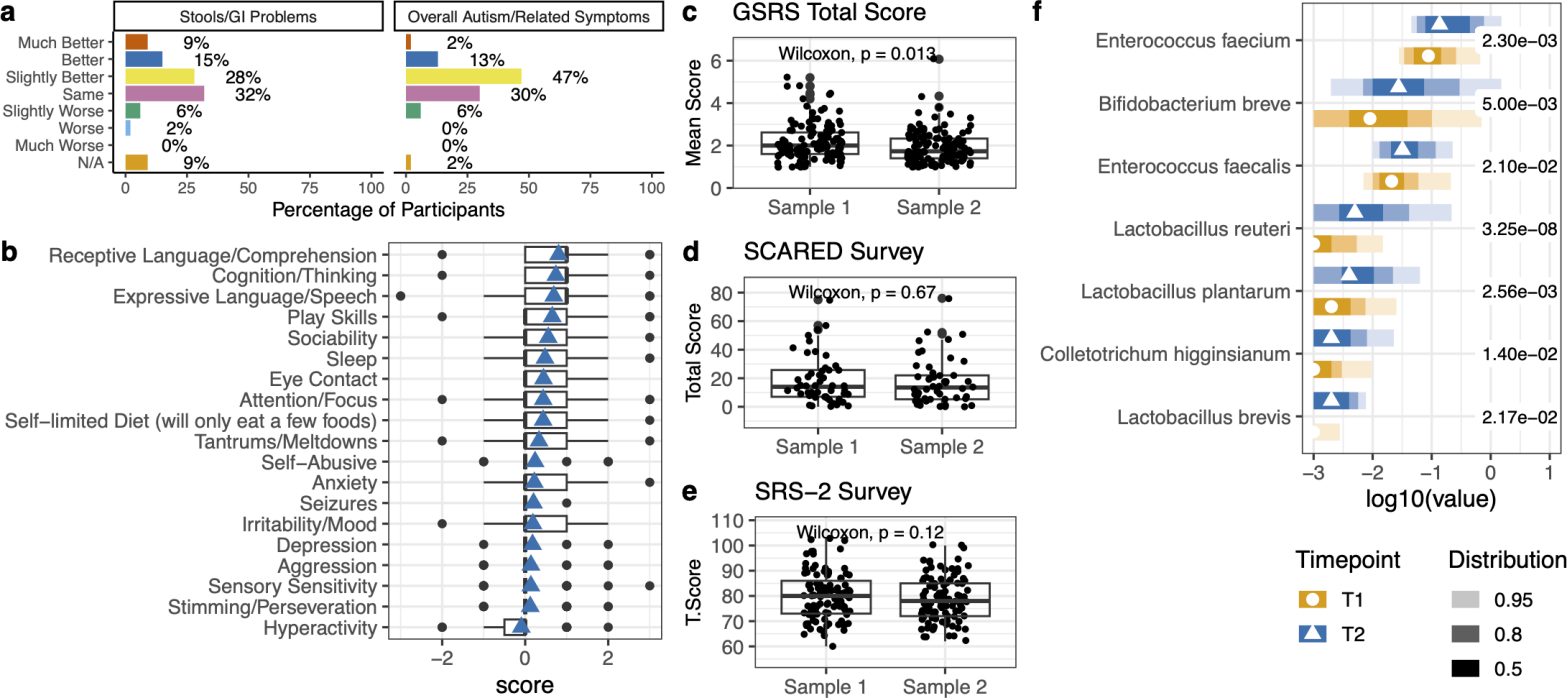
Longitudinal survey assessments. (a) PGIA timepoint 2 survey questions regarding gastrointestinal issues and overall autism-related symptoms and the proportion of the population that saw improvement, no change, or worsening symptoms. (b) The average score of the remainder of the PGIA timepoint 2 survey questions. The scale is −3 much worse, −2 worse, −1 slightly worse, 0 no change, +1 slightly better, +2 better, and +3 much better. The total or mean scores of the (c) GSRS survey, (d) SCARED survey, and (e) SRS2 surveys from paired ASD samples. Paired Wilcoxon tests were performed. (f) Of the participants who showed improvement based on the PGIA timepoint 2 survey, microbiomes were compared between timepoints at T1 (baseline) and T2. Of the top 50 microbes selected from the variables of importance from a random forest model, the listed microbes significantly differed between timepoints. Kruskal–Wallis with *post hoc* Dunn tests were performed. The points represent the median value. The shading of color along the bars indicates the distribution of the data across the *x*-axis. Of the data points, 95% of the data fall within the lightest shade, 80% of the data fall within the medium shade, and 50% of the data fall within the darkest shaded bar. The different colors represent the two cohorts. The value to the right of the bars is the adjusted *P*-value for each test.

In follow-up surveys, there was reported improvement in overall autism-related symptoms and a reduction in gastrointestinal discomfort (Fig. 7). From the PGIA survey, 62% of the subjects reported some improvement. In comparison, 30% reported no change, and 6% had slightly worsened symptoms in the question on Overall Autism and Related Symptoms (Fig. 7). There was also reported improvement by at least 50% of participants in receptive language and comprehension, expressive language and speech, cognition and thinking, and gastrointestinal problems (Fig. 7). Overall, the average PGIA score was 0.36 ± 0.55 (SD) in all surveys. In the subjects who did show improvement (positive PGIA score), the average score was 0.69 ± 0.54 (SD). The GSRS mean score significantly decreased from timepoint 1 to timepoint 2, indicating a reduction in the severity of gastrointestinal symptoms (Fig. 7). There was no significant change in the SRS2 or SCARED evaluations (Fig. 7).

Of the participants with reported improvement based on the PGIA timepoint 2 survey with scores greater than 0, we assessed microbial changes across baseline (T1) and after supplementation (T2). Probiotics *Bifidobacterium breve, Lactobacillus reuteri, Lactobacillus plantarum,* and *Lactobacillus brevis* significantly increased in proportion at timepoint 2 compared to baseline (Fig. 7). *Enterococcus faecium* and *Enterococcus faecalis* (two bacteria not used as probiotics) also increased in proportion at timepoint 2 (Fig. 7).

## DISCUSSION

As there has been growing evidence of the importance of the microbiome in the gut–brain axis and gut issues in ASD, identifying key microbial players may influence targets for therapeutic interventions. Although the relationship between the microbiome and ASD is multifactorial and complex, the goal of this study is to collect and analyze rich data on behavior, anxiety, social responsiveness, gastrointestinal symptoms, and nutritional status from ASD participants along with deep metagenomic sequencing before and after a specific microbial modulation. At baseline, there were significant differences in some microbes, pathway potentials, and gene families between the ASD and neurotypical cohorts. There were also significant differences in microbes within the ASD cohort based on social responsiveness severity (SRS2 survey) and whether participants had a gluten and dairy-free diet. After approximately 3 months of supplementation with a custom synbiotic, there was an overall improvement in gastrointestinal symptoms in the ASD cohort in 52% of participants. However, as an open-label study, it is subject to placebo effects. The follow-up surveys indicated some improvement in behavioral, cognitive, and sensory phenotypes and overall autism-related symptoms. When comparing the microbes of the participants with improvement across timepoints, there were significant increases in the proportion of probiotic species such as *Bifidobacterium breve, Lactobacillus reuteri, L. plantarum, L. brevis,* and *Enterococcus* species.

Interestingly, there was no significant difference in beta diversity by calculation with PERMNOVA in any of the data sets, and most of the differences were associated with significant alpha diversity changes. Although we did not see a significant difference between cohorts, reference (25) showed that ASD and age-matched and sex-matched controls had significant microbiome differences using PERMANOVA and differential analyses (25). Alpha diversity and the PCO axes did, however, significantly differ between our study’s cohorts. In addition, there was a significant correlation or anti-correlation between species richness and daily fruit servings, the SCARED score and microbial evenness, and PGIA and nutritional assessment scores. These results indicate that there are microbial features associated with the complex relationships between dietary habits and ASD phenotypes, and custom synbiotic supplementation can support an improvement in gastrointestinal and overall autism-related symptoms.

The finding of lower microbiome diversity in the ASD population compared to controls was consistent with some studies (20, 45, 46), but it was also contrary to others (21, 23, 47). In the De Angelis study, children aged 4–10 were from Italy. The results of the present study indicated significantly lower levels of gut microbial alpha diversity and evenness in ASD subjects compared to neurotypical age-matched children and young adults. Still, the difference was slight compared to the wide variation in alpha diversity within each group. We also found diversity differences in pathways and gene families in ASD. Heterogeneity is also seen across geographic regions, even when controlled for DNA extraction and sequencing methods (13).

Because diet contributes to microbial diversity, we investigated whether diet may be a driving force in microbial diversity in the ASD cohort. The data collected from this cohort showed no correlation between nutritional assessment and microbial diversity, which differs from what Yap et al. reported recently (19). The nutritional evaluation showed that 16.9% of the ASD cohort had excellent or very good nutrition, 21.8% had average, and 61.3% had below-average or poor nutrition. Our nutritional assessment found that the majority of children with ASD had poor diets, consistent with previous findings (23, 48). Interestingly, we found a weak negative correlation between nutritional assessment and baseline PGIA score, but it is unclear if worse ASD symptoms result in poor diet or the reverse (49, 50). Some habits and tendencies are characteristic of subjects with ASD, including aversions to specific foods and dietary intolerances (51, 52). In the 2013 Kang study, many participants had gluten and casein-free diets with nutritional supplements and probiotics (46). In the current study, approximately a third of the participants practiced casein-free, gluten-free, or gluten and casein-free diets. Additionally, nearly 65% of the ASD participants took additional nutritional supplements.

We found significant differences in microbial proportions in the participants who had a gluten and dairy-free diet compared to those who consumed gluten and dairy (Fig. 1). However, there was no significant difference in microbiome beta diversity based on a gluten-free diet. Sensitivities and behaviors may be correlated to nutritional choices. Chistol et al. found that autistic children with oral sensory sensitivity refused more types of foods and ate fewer vegetables (52).

There are similarities and differences in the gut microbiome composition of ASD found in the present study compared to the current literature. Studies have demonstrated autism gut microbiome associations with increased *Clostridiales* and *Akkermansia* and lower proportions of *Bifidobacterium*, *Prevotella*, and Firmicutes (15, 20, 21, 53–56). We found greater proportions of pathogens, including *Shigella, Klebsiella,* and *Clostridium*, and lower proportions of beneficial microbes, such as *Faecalibacterium* and *Ruminococcus,* and other beneficial microbes in ASD, similar to the results published in reference 25 from the 591 microbes associated with ASD and 169 microbes associated with their control counterparts (25) (Fig. 2). The detected pathogenic bacteria are often associated with pro-inflammatory compounds, which may be causing systemic inflammation (57, 58), while a concomitant reduction in the proportion of taxa associated with beneficial metabolisms may exacerbate symptoms (59).

Through *a posteriori* analysis, this study’s microbial functional potential signatures were consistent with the current literature on ASD and controls. Aligned with findings from Laue et al., we also found aspartate and asparagine biosynthesis superpathways to be higher in proportion in the ASD population (60). Metabolomics studies showed lower acetic acid and butyrate and a higher level of valeric acid in ASD (45). Other studies show that fecal SCFA was higher in ASD subjects than controls, specifically acetic, butyric, isobutyric, valeric, and isovaleric acids (61). However, results have also been inconclusive based on direct measurements (62). Adams et al. found lower levels of SCFA in ASD vs controls (4). While we did not directly measure SCFA in stool, metagenomic pathways of fermentation to propanoate [downstream of (S)-propane-1,2-diol degradation pathway] and acetate (downstream of purine nucleobase degradation II) SCFA were higher in ASD than in controls (Fig. 4a). Alternatively, pathways associated with butyrate production (L-lysine fermentation to acetate and butanoate, succinate fermentation to butanoate) were lower in the control population (Fig. 4c), contrary to recent findings (12). Differences in microbial associations detected in the present study compared to previously published studies may be due to differences in size and sample population, as seen in Fouquier et al. with study-site effects (13). Diet, therapies, autism severity, gastrointestinal discomfort, and other factors may also influence the differences in the gut microbiome seen across the ASD population. Stratification of the ASD population based on certain characteristics may shed light on possible specific microbial associations.

With *a priori* knowledge of specific pathways found significant in the ASD literature, we also screened those pathways in the current study. Pathways involved with sulfur metabolism, LPS, antibiotic resistance, and gamma-aminobutyric acid (GABA) significantly differed between the ASD and control populations (Fig. 4). Nirmalkar et al. found alterations in bacterial sulfur metabolism pathways after microbial transfer therapy (44). *Desulfovibrio* H_2_S and LPS production has also been associated with ASD (63, 64). Hydrogen sulfide biogenesis is attributed to the trans-sulfuration pathway, cysteine aminotransferase, or sulfate reduction (65).

Although some of these pathways were not annotated in the MetaCyc database, we investigated the superpathway of sulfate assimilation and cysteine biosynthesis and assimilatory sulfate reduction pathways associated with sulfur compound metabolism. The LPS superpathway was higher in the ASD cohort than in controls; LPS induces inflammation through the imbalance of anti-inflammatory cytokines in ASD (66). Antibiotic resistance genes were reported to be higher in children with ASD (43). One annotated antibiotic resistance pathway, polymyxin resistance, was higher in the ASD cohort (Fig. 4). Unexpectedly, peptidoglycan biosynthesis was higher in the control cohort (Fig. 4). An imbalance in GABA has also been associated with developmental delay and neurodevelopmental disorders (67, 68). We found that 4-aminobutanoate degradation was significantly lower in the ASD cohort compared to the controls (Fig. 4c).

The effect of precision synbiotic supplementation on autistic symptoms showed an overall positive response. The PGIA follow-up survey questionnaire showed an improvement in certain behaviors, and the GSRS survey showed a significant decrease in GI symptom severity for many but not all patients (Fig. 7). However, this is an open-label study, and the improvement magnitude is in the placebo effect range. We estimated the placebo effect for the PGIA by comparing it against the PGIA scores of a placebo group in a 3-month randomized, double-masked, placebo-controlled trial of a vitamin/mineral supplement for children and adults with ASD (69). The Adams et al. study used an earlier version of the PGIA, the PGI-R, which contained only 11 items on the newer PGIA. The comparison found in the Adams et al. study was that the supplement group had a PGI-R average score of 0.67 ± 0.34 and the placebo group average score of 0.34 ± 0.54 (69). The present study’s average PGIA score was 0.36 ± 0.55. Although the survey used in this study is updated compared to the Adams et al. (69) study, the PGIA score average and standard deviation may suggest that the effect of phenotypic improvement is within the placebo range (69).

The present results suggest that personalized synbiotics resulted in an overall change in the microbiome and its functional potential and may improve autism-related symptoms, including gastrointestinal symptoms. Microbial evenness was inversely correlated to the SCARED total score, such that lower evenness was associated with higher anxiety. Microbial evenness increased after supplementation at timepoint 2, but anxiety scores did not significantly improve. Preclinical and clinical studies administering prebiotics and probiotics to autistic children have also demonstrated gastrointestinal and/or symptom improvement but have used the same single ingredient or a mix of ingredients across all participants (70–77). However, a placebo-controlled study is needed to confirm changes in autism and gastrointestinal symptoms. Consideration for other surveys such as CARS-2 or DSM-V may additionally clarify these results. Impactfully, 50% or more of participants reported improvement in receptive language and comprehension, expressive language and speech, cognition and thinking, and gastrointestinal problems, which is promising and may indicate that a synbiotic supplementation is a valuable option for children and adults with ASD.

The present study design has several strengths and limitations. The strengths of this study include a metagenomic analysis of gut microbes; a large sample size; an age-matched control group of neurotypical children and adults; two timepoints for a subset of ASD participants; comprehensive behavioral, lifestyle, and dietary metadata; and a pilot open-label post-supplementation study. These characteristics are consistent with what was discussed to advance the understanding of the gut–brain axis in ASD in Morton et al. (25). The complexity of this study is the use of a different synbiotic for each participant based on their microbiome and symptoms. Limitations include the neurotypical control group having a different gender proportion to that of the ASD cohort. Although the microbiome diversity Shannon index did not differ between genders in either cohort in this study, evidence suggests differences in the microbiome between female and male subjects with ASD (78, 79). We also did not have data on the controls’ dietary habits, gastrointestinal symptoms, or behavior phenotypes. Without these data, we could not identify whether supplementation may or may not have altered the control cohort’s microbiome diversity, behavior, or gastrointestinal symptoms and compare the changes to the ASD cohort. The survey to measure improvements is comparable to a placebo effect based on the PGIA average scores. The limitations in the study, such as the lack of information on the behavioral phenotypes of the control group, may also have contributed to the placebo effect. This should be controlled for in future studies. Another limitation is the substantial drop-out rate in the study, which biases the results toward a population with greater perceived improvement.

Despite the limitations described, we did uncover potential metagenomic marker genes/pathways that may influence the improvement of autistic and gastrointestinal symptoms. Overall, revealing microbiome differences in our population enables the identification of specific targets for future therapeutic supplementation that may aid in alleviating gastrointestinal symptoms and possibly phenotypes associated with ASD. For future studies, we plan to continue investigating the longitudinal effects of synbiotic supplementation on the microbiome and phenotypes associated with ASD.

## SUPPLEMENTAL MATERIAL

Figure S1 msystems.00503-24-s0001.tiff Significant correlations between surveys and microbial diversity.

Table S1 msystems.00503-24-s0002.csv ASD T1 formulations from the 170 subjects with two timepoints.

ASM does not own the copyrights to Supplemental Material that may be linked to, or accessed through, an article. The authors have granted ASM a non-exclusive, world-wide license to publish the Supplemental Material files. Please contact the corresponding author directly for reuse.
