# Supplementary figures and images for: Precision synbiotics increase gut microbiome diversity and improve gastrointestinal symptoms in a pilot open-label study for autism spectrum disorder
Source: mSystems. 2024 Apr 25;9(5):e00503-24. doi: 10.1128/msystems.00503-24 (PMC11097633; doi:10.1128/msystems.00503-24)

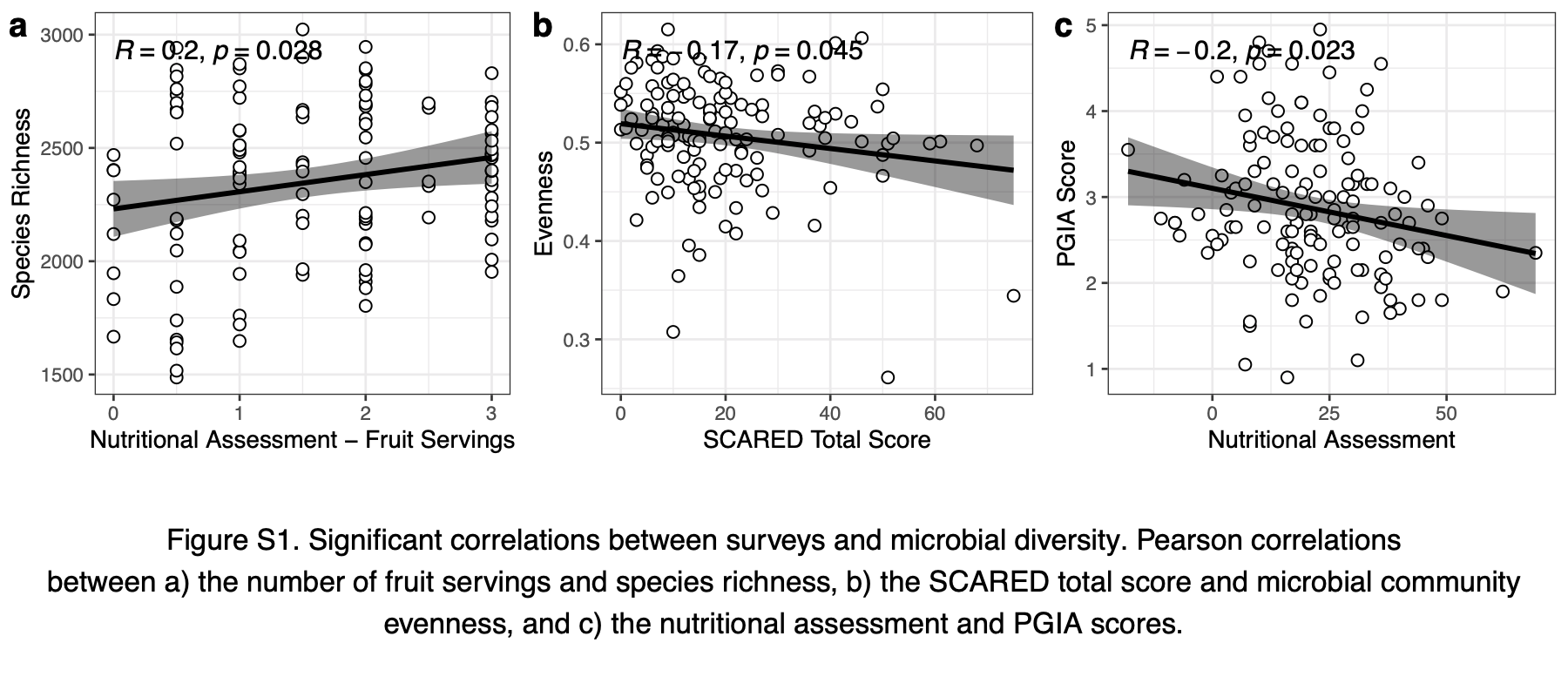

Supplement: Figure S1 — Significant correlations between surveys and microbial diversity. [file msystems.00503-24-s0001.tiff]
